# Supplementary material for: Healing of Comminuted Fractures of Long Bones in Dogs
Source: Animals (Basel). 2025 Feb 2;15(3):413. doi: 10.3390/ani15030413 (PMC11815741; doi:10.3390/ani15030413)
Supplement: Supplementary file 1 [file animals-15-00413-s001.zip › animals-3455112-supplementary.pdf]

Supplementary Material

Table S1: Contingency table of complication frequency with the number of fragments. Odds ratio (number of fragments 1–3/>3): 4.437

			Complication		
			no	yes	total
Number of fragments	1-3	Count	43	9	52
		Expected count	37.5	14.5	52.0
		% within complication	75.4 %	40.9 %	65.8 %
	> 3	Count	14	13	27
		Expected count	19.5	7.5	27.0
		% within complication	24.6 %	59.1 %	34.2 %
Total		Count	57	22	79
		Expected count	57,0	22,0	79,0
		% within complication	100.0 %	100.0 %	100.0 %

Table S2: Contingency table of complication frequency with fracture cause. In six cases, the cause of the fracture was unknown. Odds ratio (low-energy/high-energy trauma): 5.333

			Complication		
			No	yes	Total
Fracture cause	Low-energy trauma	Count	22	2	24
		Expected count	18.1	5.9	24.0
		% within complication	40.0 %	11.1 %	32.9 %
	High-energy trauma	Count	33	16	49
		Expected count	36.9	12.1	49.0
		% within complication	60.0 %	88.9 %	67.1 %
Total		Count	55	18	73
		Expected count	55.0	18.0	73.0
		% within complication	100.0 %	100.0 %	100.0 %

Table S3: Contingency table of complication frequency with open/closed fractures. Odds Ratio (closed/open): 4,082

			Complication		Total
			No	yes	
Open Fracture	No	Count	50	14	64
		Expected count	46,2	17,8	64,0
		% within complication	87,7%	63,6%	81,0%
	Yes	Count	7	8	15
		Expected count	10,8	4,2	15,0
		% within complication	12,3%	36,4%	19,0%
Total		Count	57	22	79
		Expected count	57,0	22,0	79,0
		% within complication	100,0%	100,0%	100,0%

Table S4: Complications, Treatments, and Bone Fusion Times in Osteosynthesis Cases

Abbreviations: Nr: Case number- R-radius; T-Tibia; F-Femur; H-Humerus

**Nr.**	**Age Group**	**Bone**	**Complication/Timepoint**	**Therapy**	**Fusion Proven [Weeks]**
R1	2	Radius	2 weeks: transient radial nerve paralysis	Physiotherapy	Unknown
R2	2	Radius	7 weeks: osteomyelitis	Antibiotics, Rivanol dressings	16
R3	2	Radius	--	--	20
R4	2	Radius	1 week: wound infection	Euthanasia	Unknown
R5	1	Radius	--	--	Unknown
R6	2	Radius	--	--	Unknown
R7	2	Radius	4 days: distal limb with no deep pain	Amputation	Unknown

R8	2	Radius	5 weeks: osteomyelitis + sequestrum	Debridement, sequestrum removal, autologous bone graft, antibiotics	20
R9	1	Radius	--	--	32
R10	2	Radius	9 weeks: osteomyelitis	Implant exchange (NCP -> external fixator), antibiotics	16
R11	2	Radius	--	--	Unknown
R13	2	Radius	11 days: severe wound infection	Amputation	Unknown
R14	2	Radius	--	--	Unknown
R15	2	Radius	--	--	16
R16	2	Radius	--	--	16
R17	2	Radius	--	--	16
R18	2	Radius	--	--	13
R19	2	Radius	--	--	Unknown
R20	2	Radius	--	--	16
R21	2	Radius	--	--	16
R22	2	Radius	--	--	Unknown
R23	2	Radius	--	--	16
R24	2	Radius	--	--	Unknown
F1	2	Femur	--	--	14
F3	1	Femur	--	--	9
F4	2	Femur	--	--	Unknown
F5	2	Femur	--	--	20
F6	1	Femur	3 weeks: plate break	Implant exchange (NCP), autologous bone graft	16
F7	2	Femur	--	--	5
F10	2	Femur	3 weeks: plate break; 40 weeks: plate break + nonunion	Implant exchange (3 weeks); Debridement + implant exchange (40 weeks)	52
F11	2	Femur	--	--	16

F12	2	Femur	--	--	15
F13	2	Femur	--	--	12
F14	2	Femur	--	--	Unknown
F15	1	Femur	--	--	Unknown
F16	1	Femur	--	--	11
F17	1	Femur	--	--	Unknown
F18	2	Femur	1 week: plate bending	Implant exchange (NCP)	40
F19	1	Femur	8 weeks: femur shortening, patella luxation	Correction osteotomy recommended	8
F20	2	Femur	--	--	11
F21	2	Femur	16 weeks: delayed union	Debridement, implant dynamization	28
F27	1	Femur	--	--	20
T1	2	Tibia	--	--	14
T6	2	Tibia	7 weeks: sequestrum (Tibia), delayed union	Debridement, sequestrum removal, autologous bone graft, antibiotics	24
H1	1	Humerus	--	--	5
H2	2	Humerus	--	--	13
H4	2	Humerus	2 weeks: Technovit splint loosened	Splint adjustment	8

Table S5: Complications rates in canine comminuted fractures in veterinary osteosynthesis treatments

**Study**	**Species**	**Bone(s)**	**Osteosynthesis**	**Complication Rate**	**Form**
JOHNSON, A. L. et al. (1996)	Dog	Radius/Ulna, Tibia (fracture)	External fixator	8.7% (2/23)	Delayed Union: 4.3%; Others: 4.3%
DUDLEY et al. (1997)	Dog	Tibia (fracture)	Plate, External fixator	10.6% (5/47)	Osteomyelitis: 8.5%; Nonunion: 2.1%; Technical errors: 4.2%
GUERIN et al. (1998)	Dog	Humerus (fracture)	External fixator	33.3% (2/6)	Wound infection: 16.7%; Technical errors: 16.7%; Refracture: 16.7%; Sequestrum: 16.7%
JOHNSON, A. L. et al. (1998)	Dog	Femur (fracture)	Plate	22.9% (8/35)	Osteomyelitis: 2.9%; Delayed Union: 8.6%; Technical errors: 11.4%

Table S6: Complications in veterinary osteosynthesis treatments in dogs and cats

**Study**	**Species**	**Bone(s)**	**Osteosynthesis**	**Complication Rate**	**Form**
BOONE et al. (1986)	Dog, Cat	Tibia	Various	16.4% (32/195)	Wound infection: 5.1%; Osteomyelitis: 7.2%; Nonunion: 4.1%
DURALL und DIAZ (1996)	Dog	Femur	Locking nail	20% (3/15)	Nonunion: 6.7%; Technical errors: 6.7%; Sequestrum: 6.7%
DVORAK et al. (2000)	Dog	Humerus, Radius/Ulna, Femur, Tibia	Various	11.5% (18/156)	Osteomyelitis: 1.9%; Nonunion: 1.9%; Delayed Union: 3.8%; Technical errors: 1.9%; Others: 10.9%
DUHAUTOIS (2003)	Dog, Cat	Humerus, Femur, Tibia	Locking nail	24.6% (28/114)	Wound infection: 0.9%; Nonunion: 1.8%; Delayed Union: 5.3%; Technical errors: 11.4%; Others: 6.1%
REEMS et al. (2003)	Dog, Cat	Humerus, Femur, Tibia	Plate and Rod	42.6% (20/47)	Wound infection: 2.1%; Delayed Union: 2.1%; Technical errors: 32%; Others: 6.4%

HORSTMAN et al. (2004)	Dog	Humerus, Femur, Tibia	Locking nail	45% (9/20)	Delayed Union: 5%; Technical errors: 20%; Others: 20%
HAALAND et al. (2009)	Dog	Humerus, Radius/Ulna, Femur, Tibia	Locking Compression Plate	12.8% (6/47)	Osteomyelitis: 2.1%; Technical errors: 10.6%
GUIOT und DEJARDIN (2011)	Dog, Cat	Tibia	Plate (MIPO)	13.9% (5/36)	Technical errors: 2.9%; Others: 11.4%
ALTUNATMAZ et al. (2012)	Dog, Cat	Humerus, Femur, Tibia	Intramedullary K-wire	10.9% (19/175)	Nonunion: 0.6%; Delayed Union: 1.1%; Others: 9.1%
BARNHART et al. (2013)	Dog, Cat	Humerus, Radius/Ulna, Femur, Tibia, Pelvis	Locking Compression Plate	19.4% (12/62)	Wound infection: 1.6%; Nonunion: 1.6%; Delayed Union: 4.8%; Technical errors: 18%
POZZI et al. (2013)	Dog	Radius/Ulna	Plate	6.7% (2/30)	Wound infection: 6.7%
VALLEFUOCO et al. (2015)	Dog, Cat	Humerus, Radius/Ulna, Femur, Tibia	Locking Compression Plate	17% (13/75)	Osteomyelitis: 2.7%; Delayed Union: 1.3%; Technical errors: 7%
